# Round the Hospitals

**Published:** 1919-05-17

**Authors:** 


### ROUND THE HOSPITALS.

AT the Investiture held in the Quadrangle of Buckingham Palace on May 8 the King personally conferred the Bar to the Royal Red Cross on Sister Evelyn Luard, Q.A.I.M.N.S.R., and the First Class of the Order on Sister Mary Coulson, T.F.N.S., and on Matron Helen Dalmage, Matron Jean Cameron-Smith, Matron Nella Wilson, and Sister Martha McBride, Canadian A.N.S. His Majesty conferred the Royal Red Cross, Second Class, on Sister Jean Buckingham, Sister Helen Cameron, Sister Louise Tabor, Staff Nurse Dorothy Botting, and Staff Nurse Margaret Phee, of Q.A.I.M.N.S.R.; on Matron Eliza Moseley, Sister Ellen Hall, and Sister Ada Murgatroyd, T.F.N.S.; on Matron Henrietta Barry, Assistant Matron Ruby Ingram, Sister Rosetta Nelson, and Miss Phyllis Turner, B.R.C.S.; on Sister Gertrude Donkin, Sister Edith Hibbs, Sister Sarah Johnson, Sister Bernadette Loneragan, Sister Olive MacKay, Sister Elizabeth McDougall, Sister Ethel Paynter, Sister Mabel Reynar, Sister Pauline Rose, Sister Mary Steele, Sister Martha Stewart, Sister Christine Watling,

**ROUND THE HOSPITALS—(*continued*).**

Sister Leonie Whitworth; Sister Maude Wright, and Sister Charlotte Younghusband, of the Canadian A.N.S.,; on Sister Ellen Branson, Civil Hospital Reserve. The King conferred the Military Medal, for bravery in the field, on Sister Leonora Herrington, Canadian A.N.S. Subsequent to the Investiture all the above-named members of the Military Nursing Services were received at Marlborough House by Queen Alexandra.

The King has been pleased to award the Royal Red Cross decoration to the under-mentioned ladies in recognition of their war services: Bar to Royal Red Cross, First Class—Miss. M. L. Hughes, Head Sister, Royal Naval Hospital, Plymouth; Royal Red Cross, First Class—Miss C. C. Renwick, Superintending Sister, Royal Naval Hospital, Gibraltar; Royal Red Cross, Second Class—Sister Chienside, Sister M. S. C. Gubb, Sister M. C. R. Bere, Sister B. Sandes, Sister B. M. Martin, Sister M. L. Hocking, Sister M. J. Roberts, Sister D. G. Bryant, Sister E. J. Williams, Sister M. Symonds, Sister H. F. Wells, Sister M. M. Tolhurst, Sister S. Smith, Matron E. Edwards, Matron I. Fyfe, Head Sister A. E. Skinner; Sister K. Nicholls, Sister L. A. Brooks, and Mrs. B. B. Atkinson.

Brussels has never witnessed, says the *Times* special correspondent, a more impressive spectacle than was seen at the transfer on Tuesday morning of the body of Miss Edith Cavell from the Tir National to the Gare du Nord, preliminary to its removal to England for burial at Norwich. Crowds lined the streets, and every house flew a flag at half-mast. The coffin was conveyed on a British gun-carriage, covered with a Union Jack, on which lay wreaths from the Queen of the Belgians, the City of Brussels, and Nurse Cavell's school. The bearers were British gunners. Representatives of the British, Belgian, American, and Spanish Governments had places in the procession, as well as Miss Cavell's nurses. School children lined the route throughout. The central hall at the station had been transformed into a mortuary chapel, having in the centre a tall catafalque surrounded by candles, and covered with a Union Jack draped with cloth of silver and black, on which the initial "C" was embroidered in silver. Behind Belgian troops stood nurses of the Red Cross, members of other women's corps, and representatives of the whole diplomatic, political, and social life of Brussels. The Rev. H. S. T. Gahan, British chaplain, read the opening sentences of the Burial Service, after which the coffin was conveyed by train to Ostend. On Wednesday the body was brought to Dover by H.M.S. Radiant, and in the evening was landed at the naval pier, where it was met by Rear-Admiral Dampier and General Sir Colin Mackenzie. A procession, including Red Cross and other nurses, and detachments of various women's corps, accompanied the coffin to a specially prepared chapel, where it remained pending removal to London on Thursday, and subsequently to Norwich.

To commemorate the death of Miss Edith Cavell for her country, Mr. and Mrs. Heierli, of The Hollies, West Norwood, have chosen the occasion of the funeral service at Westminster Abbey to present their house to form one of the Edith Cavell Homes of Rest for Nurses. It is situated in fine open grounds, at the moment gay with fruit blossom and spring flowers, and so sequestered that it is hard to imagine London lies almost at the gate. Nurses who for any reason are debarred from taking a long journey or can only afford a short rest will be grateful indeed.

A British Officer in Mesopotamia writes warmly in the *Times* about the services of the nursing sisters in that country. "Surely," he says, "no women have risked more than these. The graveyards tell their own story of the Englishwomen who have shared the terrors of the worst climate in the world with Englishmen. Those who have served in this country will bare their heads in reverence to those who have died and in respect for those who survive." Official reticence has hitherto concealed the names of the British nurses who lie buried in Mesopotamia. We would urge that secrecy has no longer any purpose. Let us know all about our brave country-women, how they died and where they are buried. Then let nursing sisters at home assist to raise a worthy memorial to their memory.

At last the expected Royal Warrant has been issued granting a more just and liberal rate of pay to members of the Military Nursing Services. The gratuities decided on will, we believe, give great satisfaction. The regular Nursing Service, Q.A.I.M.N.S., is placed for this purpose on a footing with regular officers. Nurses below the rank of principal matron will receive a lieutenant's gratuity, £40 for the first year's war service and £1 a month increment for each subsequent year or part of a year spent overseas, or for home service 10s. a month. Principal matrons will receive a captain's gratuity, £45, and matrons-in-chief that of a lieutenant-colonel, £75, with the same increments as the rest for subsequent service. Members of the Q.A.I.M.N.S.R. and the T.F.N.S. are to receive gratuities on the following scale: Staff nurses £20, sisters £30, and matrons £40 for the first year, and 10s. a month increment for the remainder of their service, whether at home or abroad. V.A.D. nurses and assistant nurses are to receive £10 for the first year and 10s. a month increment. Some nurses have already drawn their gratuity according to the scale previously agreed, and these may apply to the paymaster for any balance due to them on the revised rate. It would appear that for some few nurses the old scale works out to better advantage than the new.

Nurses who enrolled under a direct contract for service in connection with the Admiralty, War Office, and Air Force can now obtain the King's

**ROUND THE HOSPITALS—(continued).**

certificate of discharge on application to the headquarters of their formation. All are eligible who have served overseas in a theatre of operations with an expeditionary force between August 4, 1914, and November 11, 1918, and have been compelled to relinquish their appointments through disablement caused or aggravated by naval or military service, or directly attributable to the action of the enemy, as in air raids, etc. Some who recovered after such disablement, again volunteered and were again disabled, are eligible for the King's second certificate. Medical certificates and full particulars verified by the superior officer must be furnished by all applicants. The number of nurses eligible for both certificates is astonishingly large, and they ought not to delay making application.

---

NURSES and V.A.D. members who came to this country from overseas to take up naval or military nursing after the outbreak of war are to receive repatriation allowances at the public expense under the conditions applicable to officers. The same conditions apply to those who were serving on November 11, 1918, in hospitals under direct military control on a six months', or longer, agreement. Many overseas nurses who happened to be in this country on the outbreak of war have done fine service, and it is only just that they should be helped to get back to their own country at a time when passages are costly beyond all precedent and hard to get.

---

WE understand there are over 1,000 demobilised nurses now seeking fresh employment, and every week adds to their numbers. The nursing service is the most elastic of all careers open to women, but the process of re-absorption cannot fail to prove lengthy, and must tax the patience of many admirable nursing sisters who are fitted for high positions of responsibility. The special committees appointed to deal with Nurses' Demobilisation and Resettlement are for the moment overpowered with the magnitude of their tasks. The opportunity for securing valuable nurses ought not to be neglected by the municipal authorities. Poor Law institutions are, with but few exceptions, flagrantly understaffed and particularly so in the teaching department. Now is the time for the training-schools of various types to secure good sister tutors, with a view to raise the standard of their probationers. It may be said, without exaggeration, that there is room for good women in this capacity in training-schools throughout the country.

---

THE results of the latest examination of the Central Midwives' Board for Scotland, held on April 28 and 29, are very satisfactory. There were twenty successful candidates at the Edinburgh Centre, forty-five at the Glasgow Centre, and ten at Dundee. It would be interesting to know how many of these ladies have expressed their intention of practising as midwives.

THE *Morning Post* relates, on the authority of a "well-known London physician," an extraordinary incident. The doctor in question received a short time ago a visit from a young seamstress, whose deep-set eyes, furrowed brow, hollow cheeks, and poor dress proclaimed her deep poverty. She produced from her pocket a bundle of Treasury notes to the value of £93, which she promised to make up to £100, and desired it should be used to form a memorial for her brother who was killed in the war. She had strained every nerve to make up the money, and devoted to it all her small earnings. The strange part of the tale lies in her choice of a memorial. The money was to be used by a London hospital as an endowment for the institution of an eight-hour day. The physician does not appear to have been very successful in complying with the poor woman's request, and before he had proceeded far in the negotiations the donor died of starvation.

---

QUEEN ALEXANDRA has written to the Council of Queen Victoria's Jubilee Institute to express her satisfaction at the annual report of the Institute lately submitted to her, and particularly at the extent to which Government Departments and public authorities are invoking the aid of the Jubilee nurses in various welfare schemes. Her Majesty concludes with a sympathetic reference to "the strain put upon the members of every nursing association throughout England, Scotland, Ireland, and Wales during the past four strenuous years of war." "Everybody has done splendidly." Every nurse will feel a glow of gratification on reading Queen Alexandra's appreciation of her "untiring unselfish devotion." The patient work of the district nurses has been one of the miracles of organisation which helped to lighten the gloom of the war years.

---

NURSING arrangements are undergoing considerable improvement in the Walsall General Hospital, under a scheme recently approved by the committee. While it has not yet been found possible to inaugurate a weekly day off, there is to be a whole day off for the nursing staff once a fortnight, and this will usually follow an evening off duty, so that a maximum period of rest can be obtained. Salaries have been raised, and ward sisters are now to commence at £55, rising by annual increments of £2 10*s*. to £65. In special departments a higher rate is to be paid. Staff nurses will receive £40, £45; probationers, £20, £25, £30. These improvements will no doubt have the desired effect of making the service of the hospital more popular with candidates besides ameliorating the lot of the nurses.

---

IT is a striking sign of the times that the Holywell Board of Guardians decided at their last meeting to engage two extra assistant trained nurses with a view to the arrangement of an eight-hour day for the nursing staff. In the discussion which arose, it was elicited that the head nurse put in 66 hours a week and the staff nurses 64. The Chairman of the House Committee boldly declared

**ROUND THE HOSPITALS—(*continued*).**

that the eight-hour day would surely be made compulsory in institutions, and it was better to arrange matters on a sound basis voluntarily.

We are glad to be able to report that the Dorset County Hospital, Dorchester, has revised the scale of salaries for its nursing staff, and the off-duty time. The salaries of the ward sisters and the night sister now commence at £50 per annum, rising by yearly increments of £5 to £60; the massage sister starts at £60, and rises to £70; the staff nurses start at £45, and rise to £50; whilst the probationers' pay is £10, £15, and £20 respectively. Indoor uniform is provided, and all the nursing staff receive one day off in seven.

The services rendered by nurses in the care of permanently disabled soldiers are some of the most exacting which the war has afforded, and it is gratifying to find that the Board of Management of the British Home for Incurables have recognised this fact. They have lately presented to Mrs. Mabel Friman and Miss Agnes Johnston, a silver salver each in appreciation of their devoted care of the soldiers in the institution since 1914. It is a branch of nursing which demands the utmost skill and those rare gifts of sympathy and insight which bring the maimed and helpless patients back from despair to a living trust in their destiny.

The maternity home recently opened at Plymouth in connection with the Three Towns Nursing Institution is an excellent specimen of the institutions now being founded all over the country for the benefit of working mothers. No town needs provision of this kind more acutely than Plymouth, where there are acres of slums in which child-birth cannot but be considered a precarious adventure. It is hoped to add to the home a hostel where, in special cases, the children of poor mothers can be taken care of during the lying-in period. The rooms are bright and airy, and the home shows evidence already of becoming thoroughly popular with those for whom it is intended. It will greatly strengthen the hands of the nurses.

At the annual meeting of the Paddington and St. Marylebone District Nursing Association, which was held at Lady St. Helier's house in Portland Place, a falling-off in the number of cases was reported and attributed to an improvement in the general health of the community, arising out of higher wages. It is the more remarkable since the period covers the influenza epidemic. The increasing care and vigilance of district nurses has thus already reaped a high reward and we believe Lady St. Helier to have spoken no more than the truth, in declaring that it was entirely due to the skill and zeal of the nurses that many infectious illnesses in the military hospitals had affected the civil populations so little. There should be no delay in wiping off the reproach to these wealthy boroughs of allowing this valuable organisation to close its year with a deficit of £100.

The fall in food prices recorded to date is stated to be much sharper than the average rise was during the war. The statistics quoted in the *Labour Gazette* show that during the month of February alone the average level had fallen from 130 per cent. to 120 per cent. above the pre-war standard. Hospital managers will need to be on the alert to take the utmost advantage of changing prices. This is no time for accumulating reserves. The moment, however, has hardly yet come for finding the fall in prices reflected in the weekly expenditure. Every one has been on short commons. The cook has had little chance for the last three years of distinguishing herself. Now that food is more plentiful the institution table ought to be better furnished. Little luxuries, such as dates, which give good food value at 6d. a lb., may begin to reappear. More sugar can be allowed for puddings and cakes, more fats for cooking and general consumption. These relaxations of war fare will wipe out economies effected in the purchase of supplies. But if they bestow an improved sense of *bien être* on the whole household and increase the common stock of energy they will be worth the money.

Has embroidery come to stay as a feature in the treatment of disease? It would seem that for long chronic cases, in sanatoria and the like, it had proved its value in rousing men from inertia, and it would doubtless prove not less efficacious in female chronic wards. All that is wanted is the continued ministrations of the many practical teachers who introduced the art into military hospitals. Why does the soldier lose all his attractiveness for the outside public as soon as he is discharged and no longer wears the blue? He is the same fine fellow after as before, and even more in need of friendship, but matrons of the special hospitals where he is to be found are, alas, by no means overwhelmed with offers of help on his behalf.

An excellent account is given in the April number of the *American Journal of Nursing* of the revolution effected in Californian hospitals by the inclusion of hospital students in the 1913 Eight-Hour Law. The law reads that no female shall be employed in any hospital for more than eight hours during any one day, or more than forty-eight hours in any one week. It does not apply, however, to graduate nurses in hospitals. Considerable confusion followed the enactment of the law, and much hardship was at first experienced both by patients and by probationers themselves owing to lack of preparation or organising ability in the authorities. By degrees things settled down, and we learn that many superintendents who were strongly opposed at first are now quite satisfied with the results and would oppose the repeal of the law. Admissions to the training-schools have increased, and women of good qualifications are no longer discouraged from making application. The general health index of the students has perceptibly risen, and they find themselves in better physical condition at the end of the course than at the beginning.

